# The Screening and COnsensus Based on Practices and Evidence (SCOPE) Program–Results of a Survey on Daily Practice Patterns for Patients with mCRC

**DOI:** 10.3390/curroncol28030194

**Published:** 2021-06-04

**Authors:** Gerald Prager, Claus-Henning Köhne, Juan Manuel O’Connor, Fernando Rivera, Daniele Santini, Harpreet Wasan, Jean Marc Phelip

**Affiliations:** 1 Department of Medicine, Medical University of Vienna, 1090 Vienna, Austria; 2 Innere Medizin–Onkologie und Hämatologie, Klinikum Oldenburg, 26133 Oldenburg, Germany; koehne.claus-henning@klinikum-oldenburg.de; 3 Department of Clinical Oncology, Instituto Alexander Fleming, Buenos Aires C1426ANZ, Argentina; juanmanuel.oconnor@gmail.com; 4 Department of Medical Oncology, University Hospital Marqués de Valdecilla, IDIVAL, 39008 Santander, Spain; fernando.rivera@scsalud.es; 5 Department of Medical Oncology, Policlinico Universitario Campus Bio-Medico Rome, 00128 Rome, Italy; d.santini@unicampus.it; 6 Department of Cancer Medicine, Hammersmith Hospital, London SW7 2AZ, UK; h.wasan@imperial.ac.uk; 7 Department of Hepato-Gastroenterology, University Hospital of Saint-Étienne, 42270 Saint-Étienne-Priest-en-Jarez, France; j.marc.phelip@chu-st-etienne.fr

**Keywords:** metastatic colorectal cancer, practice patterns, trifluridine-tipiracil, regorafenib, *KRAS*-mutated mCRC, *KRAS*-wildtype mCRC

## Abstract

The SCOPE project aimed to better understand practice patterns, identify drivers for treatment goals, and determine third- and fourth-line treatment choices for patients with metastatic colorectal cancer (mCRC). The survey was developed by an expert panel of gastrointestinal oncologists. Questions concerned general practice patterns, and treatment decisions for three hypothetical patient case scenarios. Participants had to routinely manage patients with mCRC. We present results from 629 participants who provided input on patient treatment scenarios (data cutoff: 17/01/2020). Prolonging overall survival (OS; 51%) was the main aim in first line. In third line, quality of life (QOL) was the primary goal (34%). Forty-three percent also cited efficacy-focused goals; 18% and 13% noted prolonging OS and improving progression-free survival as main aims, respectively. For fit and active patients, 89% of respondents considered trifluridine-tipiracil an appropriate third-line treatment; regorafenib (31%) or clinical trial enrollment (29%) were the fourth-line options. For patients with comorbidities and limited caregiver support, trifluridine-tipiracil was the preferred third-line treatment (70%). For *KRAS*-mutated patients with comorbidities and adverse events who received prior oxaliplatin, 90% considered oxaliplatin rechallenge an unsuitable third-line treatment, mainly due to the risk of cumulative toxicity (75%). In the third/fourth-line settings, trifluridine-tipiracil followed by regorafenib was the most common option (54%); 17% chose regorafenib followed by trifluridine-tipiracil. Efficacy coupled with QOL are important goals in third-line treatment. Daily practice patterns reflect the guideline recommendations in third- and fourth-line settings, with a trend toward using trifluridine-tipiracil versus regorafenib in *KRAS*-wildtype and *KRAS*-mutant tumors.

## 1. Introduction

Colorectal cancer (CRC) is one of the most commonly diagnosed cancers, accounting for approximately 10% of cancer-related deaths globally/worldwide [1]. Much progress has been made in combating this disease in recent years through early detection and the rise of therapeutic options. This has led to substantial improvements in overall survival (OS), although survival rates among patients with metastatic disease still remain suboptimal, with an overall 5-year survival rate of <15% for patients with stage IV cancer [2].

CRC is a highly heterogeneous disease that has a number of genetic and epigenetic drivers. Mutations in oncogenes, such as *KRAS*, *NRAS*, *BRAF*, and *PIK3CA*, result in the dysregulation of key signaling pathways, thereby promoting progression [3]. Such diversity means there is no “catch-all” molecular therapy, and an array of therapeutic options have become available in recent years. While the existence of such an armamentarium has led to a decrease in mortality [4,5,6,7,8], it has also complicated the therapeutic decision-making process.

When deciding on the optimal management strategy, a number of factors must be taken into account. It is important to identify the mutation profile of patients with driver mutations, as it can be predictive of how a patient responds to treatments, e.g., epidermal growth factor receptor (EGFR)-targeted therapies. In addition, properties such as tumor sidedness and existing comorbidities, as well as more practical topics like reimbursement policies and distance from hospital, all impact treatment choice.

A number of national and international guidelines, such as those of the National Comprehensive Cancer Network and the European Society for Medical Oncology [9,10], exist to guide physicians toward optimal therapeutic choices. The current third-line and fourth-line therapy recommendations for patients with metastatic (m)CRC include anti-EGFR antibody with or without irinotecan [9,10]. For those with *RAS*-wildtype tumors who have received EGFR inhibitors or are in the rechallenge setting, trifluridine-tipiracil or regorafenib are recommended; both drugs are also recommended in patients with *RAS*-mutated mCRC in the third- and fourth-line settings [9,10]. In certain countries, such as Spain and the United States, trastuzumab plus pertuzumab or lapatinib may be used in patients with human epidermal growth factor receptor 2 (*HER2*)-amplified tumors [9,11], and immune checkpoint inhibitors, including nivolumab and ipilimumab, are approved in the United States and recommended by guidelines for patients with deficient mismatch repair/microsatellite instability (MSI)-high tumors [9]. Best supportive care may also be considered, depending on Eastern Cooperative Oncology Group performance status, and in those patients for whom all other therapies have failed. However, information about adherence to these guidelines and real-world practice patterns is lacking. Moreover, as guidelines are based on the results of randomized controlled trials, there may be a gap between the efficacy reported in clinical trials and that observed in real-world daily practice, and this may impact adherence.

Herein, we describe the results of the Screening and COnsensus based on Practices and Evidence (SCOPE) program, which was designed to gather real-word insights in current clinical practices for the management of patients with mCRC who have received prior treatment. The main objectives of this program were to: understand the current clinical practice in mCRC in third and subsequent lines of treatment; identify drivers for treatment choices (practitioner, patient, and tumor characteristics); describe geographic differences between countries; and contribute to consensus-seeking on key clinical questions for mCRC pretreated patients.

## 2. Materials and Methods

The survey was developed independently by an expert panel of gastrointestinal oncologists from different countries who sought to understand more about the current real-world practice patterns of their peers. Healthcare providers were eligible to participate if they were personally responsible for, and actively involved in, the management of patients with mCRC. Physicians completed a declarative questionnaire section (Table S1) and gave input on three hypothetical patient cases representing three different treatment scenarios in the third and fourth line. Ethics approval was not required as no patients were involved in this program.

The three patient cases were designed to be as relevant as possible to real-world practice, and were developed through an iterative process by the expert panel. Initially, the expert panel identified three treatment scenarios that were frequently encountered in their practice and that posed challenges with regard to the various available treatment options. The experts then discussed and reviewed each of the cases multiple times to refine the hypothetical patients’ treatment histories and ensure that the treatment options and respective dosages were representative of those seen in their clinical practice.

A brief description of the three patient cases follows, and a fuller description including each patient’s treatment history can be found in Figure S1 [4].

- Case 1: A fit and active 54-year-old male with a left-sided, *KRAS*-wildtype colon adenocarcinoma
- Case 2: A 68-year-old female with a *KRAS*-mutated left-sided colon adenocarcinoma, comorbidities, and previous tolerability issues
- Case 3: An 82-year-old male with a *KRAS*-wildtype right-sided colon adenocarcinoma who had comorbidities, limited support, and difficult hospital accessibility

Data were collected from physicians at in-person meetings. Each of the cases were presented to the delegates by the meeting moderator in the respective countries. Answers to the questions posed were collected via a Web application made available to each of the participants during the meetings, which enabled anonymization of data to reduce any bias in responses. Multivariate regression and clustering analyses were conducted to examine whether the combined effect of different parameters influenced prescribing behavior.

## 3. Results

Data were collected from 87 meetings, conducted in 12 countries, with a total of 706 participants. All of the meetings were undertaken between November 2018 and January 2020, with a final data cutoff date of 17 January 2020. The final analysis included only participants who provided input on patient treatment scenarios (*N* = 629), and did not include the nurses and pharmacists who also participated in the meetings. Participant demographics are summarized in Table 1; the majority were medical oncologists (69%), practiced in university hospitals (47%), and were 35–55 years old (58%).

### 3.1. Practice Patterns

*KRAS, PIK3CA, BRAF*, and MSI testing were frequently requested for patients with mCRC. Systematic *KRAS* and *NRAS* testing was undertaken by 91% of physicians. In total, 77% of physicians requested *BRAF* tests; when analyzing the results by country, the rate of *BRAF* testing was slightly lower (69%) in Central/Eastern Europe compared with the overall uptake. The rates of systemic MSI testing were lower, with 58% of physicians routinely requesting this test. Screening for HER2 was not routinely performed, with only 11% of physicians requesting this test.

**Figure 1:**
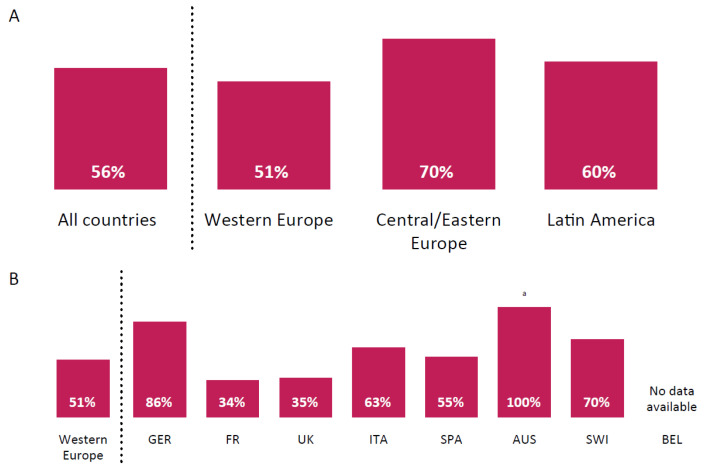
Percentage of physicians who consider tumor sidedness when making treatment decisions for patients with *KRAS* wildtype across (**A**) different regions and (**B**) Western Europe. AUS = Austria; BEL = Belgium; FR = France; GER = Germany; ITA = Italy; SPA = Spain; SWI = Switzerland; UK = United Kingdom. ^a^ Small sample size, so caution should be taken when interpreting these data. Schemes follow the same formatting.

Physicians were also questioned about the impact of tumor sidedness on their first-line targeted therapy choice for patients with *KRAS* wildtype. The degree to which tumor sidedness influenced physicians’ first-line targeted therapy choice for patients with *KRAS* wildtype varied across geographic regions. More physicians in Central/Eastern Europe (70%) than in Western Europe (51%; Figure 1A) considered tumor sidedness when making treatment decisions. There was also variation in the physicians’ responses between the different countries in Western Europe; for example, 86% of German practitioners consider tumor sidedness when determining the optimal treatment strategy versus only 34% of practitioners in France (Figure 1B).

**Figure 2:**
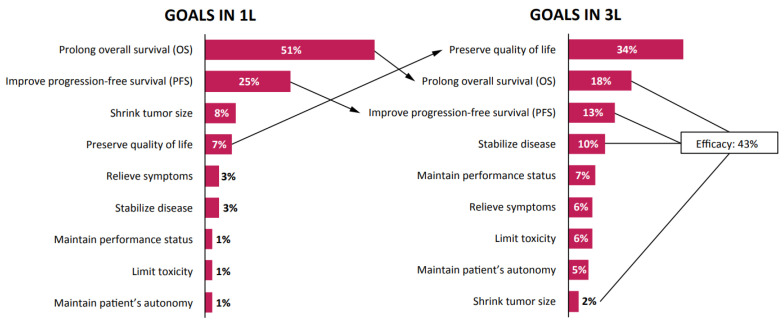
Treatment goals in the first-(1L) and third-line (3L) settings.

Physicians’ treatment goals for patients with mCRC differed between the first and third lines (Figure 2), although the primary goals in each setting were homogeneous across the different countries. In the first line, prolonging OS was the primary goal (51%), followed by improving progression-free survival (PFS; 25%); these results were consistent regardless of the region or country. In the third-line setting, preserving quality of life (34%) was the most common primary goal, although participants also considered efficacy goals, such as prolonging OS (18%), PFS (13%), stabilizing disease (10%), and shrinking tumor size (2%), to be relevant. Taken together, efficacy objectives accounted for 43% of responses.

### 3.2. Patient Cases

Case 1: A fit and active 54-year-old male with a left-sided, *KRAS*-wildtype colon adenocarcinoma

**Figure 3:**
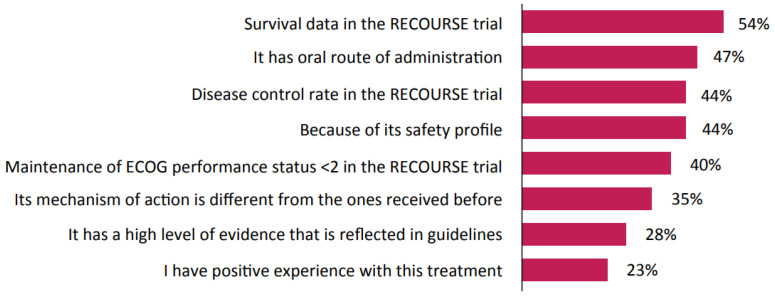
Physicians’ responses on why oxaliplatin rechallenge is an unsuitable third-line treatment in a *KRAS*-mutant patient with comorbidities and previous tolerability issues. ECOG = Eastern Cooperative Oncology Group.

Participants were asked whether they considered trifluridine-tipiracil an appropriate third-line treatment choice for a fit and active patient with *KRAS*-wildtype mCRC who received first-line anti-EGFR and second-line anti-vascular endothelial growth factor; 89% considered trifluridine-tipiracil to be a suitable therapy for this patient. Physicians were then asked to specify from a list of responses (where more than one response could be chosen) the factors that drove their choice. The survival and safety data from the RECOURSE trial [4] were found to be among the key drivers, in addition to the oral route of administration of trifluridine-tipiracil (Figure 3). Of the 11% who considered trifluridine-tipiracil inappropriate for this patient, the principal reason was the desire to rechallenge with one of the prior therapies. When asked about the preferred fourth-line treatment choice after receiving trifluridine-tipiracil for this patient, 31% choose regorafenib as their preferred option, while 29% cited enrollment in a clinical trial. These results were consistent with national and international guidelines.

Case 2: A 68-year-old female with a *KRAS*-mutant left-sided colon adenocarcinoma, comorbidities, and previous tolerability issues

**Figure 4:**
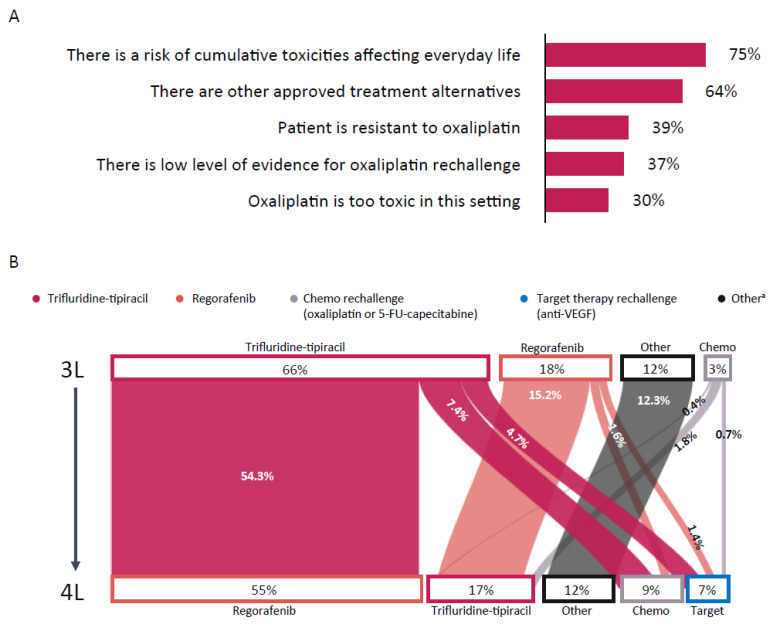
(**A**) Physicians’ responses on why oxaliplatin rechallenge is an unsuitable third-line treatment in a *KRAS*-mutant patient with comorbidities and previous tolerability issues, and (**B**) physicians’ responses on the preferred treatment strategy for a patient with *KRAS*-mutant tumor, comorbidities, and previous tolerability issues. 3L = third line; 4L = fourth line; 5-FU = 5-fluorouracil; VEGF = vascular endothelial growth factor. ^a^ Defined as unspecified.

The suitability of oxaliplatin rechallenge as third-line treatment in a patient with *KRAS*-mutated mCRC, who also presented with controlled hypertension and controlled type 2 diabetes (without neuropathy), was assessed. In total, 90% of physicians deemed such a treatment inappropriate for this patient. The key drivers for their choice were the risk of cumulative toxicity (75%), and the availability of alternative approved treatment options (64%) (Figure 4A). A total of 54% of physicians considered trifluridine-tipiracil followed by regorafenib the preferred third/fourth-line treatment sequence for this patient (Figure 4B).

Case 3: An 82-year-old male with a *KRAS*-wildtype right-sided colon adenocarcinoma who has comorbidities, limited support, and difficult hospital accessibility

Participants considered trifluridine-tipiracil the most suitable treatment option for an elderly patient with a *KRAS*-wildtype tumor who has hypertension and type 2 diabetes, previous side effects (neuropathy, rash, diarrhea, and stomatitis), limited caregiver support, and hospital accessibility issues, with 70% citing this as their preferred treatment option.

No statistically significant associations were observed when conducting the multivariate regression and clustering analyses to see if any correlations could be made between the various demographic characteristics of the participants and the prescribing behaviors reported for each of the cases.

## 4. Discussion

The SCOPE program provided real-world insights from over 600 physicians in 12 different countries regarding the management strategies that were being used in 2019 for patients with mCRC. As would be expected, molecular testing was routinely performed in patients to determine the optimal therapy, although some regional differences were observed. For example, while testing for *KRAS* and *NRAS* mutations was common practice for almost all physicians, the uptake of *BRAF* testing was not quite so homogeneous, with fewer physicians in Central/Eastern Europe undertaking this particular molecular testing. This could be due, in part, to logistic factors such as differences in pharmaceutical expenditures and reimbursement policies across European countries [12], which could impact physician decisions regarding mutation testing. It may also reflect the lower prevalence of *BRAF* mutations in patients with mCRC (10%) [13] compared with the higher incidence of *KRAS/NRAS* mutations (56%) [14].

Regional differences were also observed concerning the impact of tumor sidedness on treatment decisions for patients with mCRC and *KRAS* wildtype. Physicians in Central/Eastern Europe were more likely to consider tumor sidedness when determining the optimal treatment strategy, compared with physicians in Western Europe or Latin America. Interestingly, there was also notable variation regarding the importance of tumor sidedness within Western European countries. The majority of physicians in Germany, Switzerland, and Italy factored tumor sidedness into their treatment decisions, while only a portion of physicians in France and the United Kingdom considered this characteristic relevant. These differences may reflect the lack of guidance in the 2016 European Society for Medical Oncology (ESMO) guidelines regarding primary tumor location and its impact on response to certain treatments [10]. Originally, there was some controversy surrounding the significance of tumor sidedness, with conflicting studies reporting that this factor may [15,16] or may not [17] be a prognostic indicator for mCRC treatment outcome. However, following the publication of a large meta-analysis in 2017 [18], tumor sidedness began to be recognized as an important predictive and prognostic factor. In particular, the location of the primary tumor is now known to affect the outcomes of EGFR-targeted therapies, with left-sided tumors seemingly responding better [19]. That said, the ESMO 2016 guidelines predate these publications; hence, it is understandable that physicians across Europe may weigh this feature differently on the basis of their own experiences and patient populations. The observed variations may also be accounted for by the differences in reimbursement seen across these countries.

The responses regarding the main treatment goals in the first line and the third line were homogeneous across the different countries. Unsurprisingly, in first-line treatment, efficacy was the main aim of treatment, with 51% of participants wanting to prolong OS. Interestingly, when it came to the main objectives in the third-line setting, the overall goals of efficacy versus quality of life were well balanced, with 57% of physicians prioritizing quality of life and 43% prioritizing efficacy. The importance that physicians placed on quality of life goals reflects the growing realization that the impact of treatment on quality of life should be a key consideration when developing treatment plans, particularly in the third- and fourth-line settings [20]. Such factors are a critical component in a comprehensive treatment plan and should be discussed with the patient when management strategies are being developed.

The ESMO guidelines recommend trifluridine-tipiracil or regorafenib for all patients in the third-line setting, regardless of their mutation status (*RAS* wildtype or mutated, or *BRAF* wildtype or mutated) or previous treatments. For patients who have *RAS*-wildtype and *BRAF*-wildtype mCRC and who have not received prior anti-EGFR therapies, the ESMO also recommends cetuximab or panitumumab [10]. Similarly, the National Comprehensive Cancer Network endorses trifluridine-tipiracil or regorafenib in the third-line setting, as well as recommending cetuximab or panitumumab, preferably in combination with irinotecan, for patients who are EGFR antibody treatment naive [9]. The results from our survey demonstrate that physicians’ treatment decisions are largely aligned with current international guidelines for mCRC treatment, with physicians opting to use trifluridine-tipiracil and regorafenib as third- and fourth-line treatment choices. Our data also show that trifluridine-tipiracil is the preferred third-line treatment option for patients with mCRC regardless of their age, *KRAS* mutation status, tumor sidedness, comorbidities, or previous tolerability issues. The survival data from the RECOURSE trial [4] were the primary driver for recommending third-line trifluridine-tipiracil treatment, although other factors including route of administration, disease control rate, favorable safety profile, and mechanism of action may have also contributed to physician treatment decisions. In addition to mCRC mutation status, additional factors such as patient fitness, comorbidities, and previous tolerability issues were also seen to influence physician treatment decisions. It is possible that logistic issues including drug availability and reimbursement policies could also impact physician treatment choices [12].

Trifluridine-tipiracil followed by regorafenib is the preferred sequence of treatment in the third and fourth lines for patients with *KRAS*-wildtype mCRC who have received prior anti-EGFR therapy. While current treatment guidelines recommend both trifluridine-tipiracil and regorafenib in the third- and fourth-line treatment settings, there is no guidance regarding the optimal treatment sequence of these two agents in later treatment lines. Our data suggest that physicians have their own preferences for treatment sequencing, but prospective clinical trials are required to further validate the optimal sequence.

## 5. Conclusions

The SCOPE program has provided enhanced insight into real-world treatment practices for patients with mCRC and highlights how logistic, medical, and geographic factors may contribute to physician treatment decisions. Our data show that efficacy, coupled with quality of life, are important goals in the third-line setting. This is reflected in the preferred third- and fourth-line treatment choices for patients with mCRC. The daily practice patterns revealed by this survey reflect the recommendations of several international guidelines [9,10] on the use of the two approved treatments in the third- and fourth-line settings. Finally, our data suggest there is a trend toward the use of trifluridine-tipiracil followed by regorafenib in patients with *KRAS*-wildtype and *KRAS*-mutant tumors.

## Figures and Tables

**Table 1:** Demographics of participants (final-analysis population).

Characteristic, %	*N* = 629
Age, yr	
<35	28
35–55	58
>55	13
Medical specialty	
Medical oncologist	69
Radio-oncologist	14
Gastroenterologist	16
Surgeon	1
Type of practice setting ^a^	
Private office	8
University hospital	47
Anticancer center	21
General/Public hospital	18
Private hospital/clinic	7
Patients with mCRC/month	
≥40	19
30–39	10
20–29	22
10–19	31
<10	18
Number of participants per country	
Argentina	47 (7.5)
Austria	24 (3.8)
Belgium	6 (1.0)
Croatia	29 (4.6)
France	130 (20.7)
Germany	58 (9.2)
Hungary	74 (11.8)
Italy	53 (8.4)
Slovenia	12 (1.9)
Spain	31 (4.9)
Switzerland	40 (6.5)
United Kingdom	125 (19.9)

mCRC = metastatic colorectal cancer; yr = year. ^a^ Main setting.

## Data Availability

The data presented in this study are available on request from the corresponding author. The data are not available in a public repository due to their non-clinical nature.

## Supplementary Materials

The following are available online at https://www.mdpi.com/article/10.3390/curroncol28030194/s1, Figure S1: Disease characteristics and treatment history of the three patient cases, Table S1: Declarative questions of the survey.
