# Supplementary material for: The Screening and COnsensus Based on Practices and Evidence (SCOPE) Program–Results of a Survey on Daily Practice Patterns for Patients with mCRC
Source: Curr Oncol. 2021 Jun 4;28(3):2097–106. doi: 10.3390/curroncol28030194 (PMC8293190; doi:10.3390/curroncol28030194)
Supplement: Supplementary file 1 [file curroncol-28-00194-s001.zip › curroncol-1248688_SupplementalMaterial/Figure S1.pdf]

CASE 1

A fit and active 54-year-old male with a left-sided, *RAS*-wildtype colon adenocarcinoma

**Anton**	54-year-old male<br>Lives close to hospital<br>Plays tennis and violin
**Comorbidities**	None
RAS	Wildtype

BRAF	Wildtype
**MS status**	MS stable
**Condition**	Left-sided colon adenocarcinoma
**ECOG PS**	0

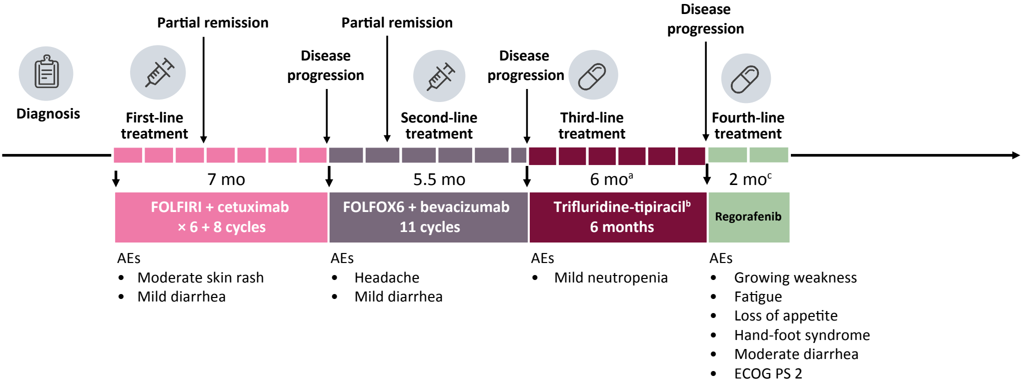

AE = adverse event; ECOG PS = Eastern Cooperative Oncology Group performance status; FOLFIRI = folinic acid, fluorouracil, irinotecan; FOLFOX6 = folinic acid, fluorouracil, oxaliplatin; MS = microsatellite; PFS = progression-free survival.

[a]In clinical trials, reported median PFS = 2.0 months (Mayer RJ, et al. N Engl J Med 2015;372:1909–1919). [b]Starting dose is 35 mg/m^2 administered orally twice a day on days 1–5 and days 8–12 of each 25-day cycle until progression or unacceptable toxicity. [c]Clinical trials of regorafenib report median PFS = 1.9 months (Grothey A, et al. Lancet 2013;381:303–312).

CASE 2

A 68-year-old female with *KRAS*-mutated left-sided colon adenocarcinoma, comorbidities, and previous tolerability issues

**Maria**	68-year-old female<br>Lives close to hospital<br>Lives with grandchildren and family
**Comorbidities**	Controlled hypertension, controlled type 2 diabetes without signs of neuropathy

RAS	*KRAS* mutant
BRAF	Wildtype
**MS status**	MS stable
**Condition**	Left-sided colon adenocarcinoma
**ECOG PS**	1

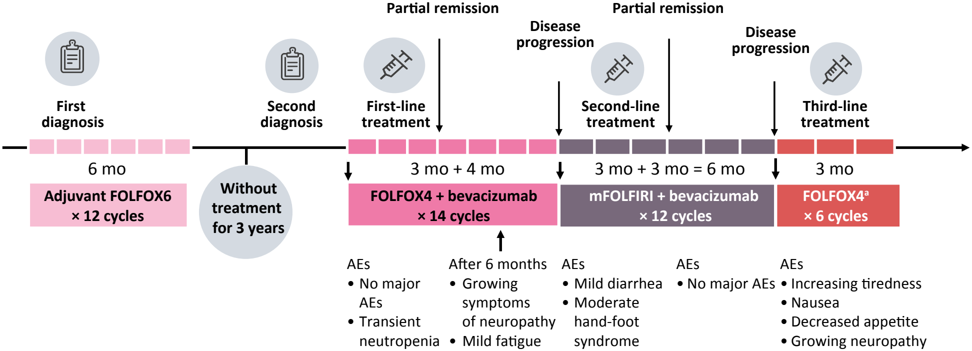

5-FU, 5-fluorouracil; AE = adverse event; ECOG PS = Eastern Cooperative Oncology Group performance status; FOLFOX4 = folinic acid, fluorouracil, oxaliplatin; IV, intravenous; mFOLFIRI = simplified LV5FU2 + irinotecan; MS = microsatellite; q, every; w, week.

Irinotecan: 180 mg/m^2 once every 2 weeks IV infusion over 30–90 minutes.

Bevacizumab: 10 mg/kg IV infusion over 90 minutes, day 1.

FOLFOX4 + bevacizumab × 6 cycles = day 1: oxaliplatin 85 mg/m^2 and leucovorin 200 mg/m^2 concurrently IV, then 5-FU, 400 mg/m^2 IV bolus, followed by 600 mg/m^2 continuous IV; day 2: leucovorin 200 mg/m^2 IV, then 5-FU 400 mg/m^2 IV bolus, followed by 600 mg/m^2 continuous IV; repeated q2w + bevacizumab. 10 mg/kg IV infusion over 90 minutes/day.

[a]Other indicated and recommended treatment options are trifluridine-tipiracil and regorafenib.

CASE 3

An 82-year-old male with *RAS*-wildtype right-sided colon adenocarcinoma who has comorbidities, limited support, and difficult hospital accessiblity

**Carlos**	82-year-old male<br>Lives alone<br>Active traveler
**Comorbidities**	Controlled hypertension, controlled type 2 diabetes

RAS	Wildtype
BRAF	Wildtype
**MS status**	MS stable
**Condition**	Right-sided colon adenocarcinoma
**ECOG PS**	1

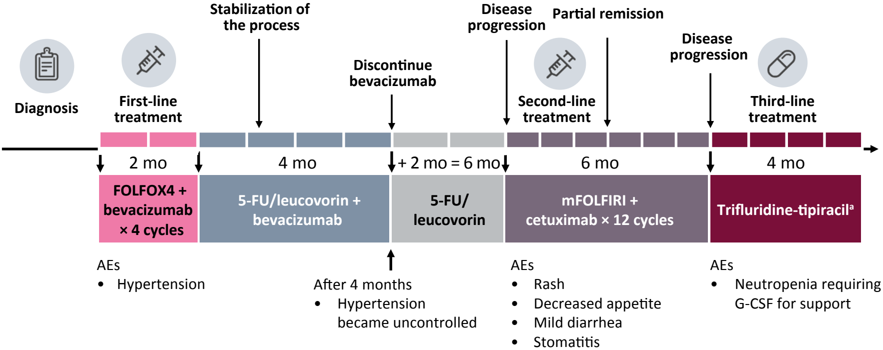

5-FU, 5-fluorouracil; AE = adverse event; ECOG PS = Eastern Cooperative Oncology Group performance status; FOLFOX4 = folinic acid, fluorouracil, oxaliplatin; G-CSF = granulocyte colony-stimulating factor; IV, intravenous; mFOLFIRI = simplified LV5FU2 + irinotecan; MS = microsatellite.

FOLFOX4 + bevacizumab × 4 cycles = day 1: oxaliplatin 85 mg/m^2 and leucovorin 200 mg/m^2 concurrently IV, then 5-FU 400 mg/m^2 IV bolus followed by 600 mg/m^2 continuous day 2: leucovorin 200 mg/m^2 IV, then 5-FU 400 mg/m^2 IV bolus, followed by 600 mg/m^2 continuous IV; repeated every 2 weeks + Bevacizumab 10 mg/kg IV infusion over 90 minutes, day 1.

mFOLFIRI + cetuximab: irinotecan administered at a dose of 180 mg/m^2 every 2 weeks with combinations of infusional 5-FU–folinic acid (LV) + IV cetuximab weekly (400 mg/m^2 as a 120-minute infusion at first week, then 250 mg/m^2 as a 60-minute IV infusion).

Other indicated and recommended treatment option in third line is regorafenib.

[a]Starting dose 35 mg/m^2 administered orally twice a day on days 1–5 and days 8–12 of each 28-day cycle until disease progression or unacceptable toxicity.

[b]In clinical trials, reported median PFS = 2.0 months (Mayer RJ, et al. N Engl J Med 2015;372:1909–1919).
