# Supplementary material for: The Screening and COnsensus Based on Practices and Evidence (SCOPE) Program–Results of a Survey on Daily Practice Patterns for Patients with mCRC
Source: Curr Oncol. 2021 Jun 4;28(3):2097–106. doi: 10.3390/curroncol28030194 (PMC8293190; doi:10.3390/curroncol28030194)
Supplement: Supplementary file 1 [file curroncol-28-00194-s001.zip › curroncol-1248688_SupplementalMaterial/Table S1.docx]

**Supplementary Materials**

| **Table S1.** Declarative questions of the survey. | |
| --- | --- |
| **Question** | **Response options** |
| What is your primary medical specialty? | Medical oncologist  Radio-oncologist  Gastroenterologist  Surgeon |
| What type of practice do you mainly work in? | Private office/Private focus office  Teaching hospital/University hospital  Cancer center/Oncologic reference center  General/Non-university public hospital  Private hospital/Private clinic |
| On average, how many patients with metastatic colorectal cancer do you manage in total in a typical month? | <10  10–19  20–29  30–39  ≥40 |
| How old are you? | <35 years old  36–55  >55 |
| Which of the following tests do you request for your mCRC patients?^a^ | *KRAS* and *NRAS*  *KRAS*  *BRAF*  MSI  HER2 |
| In general, what are your treatment goals in first-line mCRC?^b^  What are your first-line treatment drivers?^b^ | Improve progression-free survival  Limit toxicity  Maintain patient’s autonomy  Maintain performance status  Preserve quality of life  Prolong overall survival  Relieve symptoms  Shrink tumor size  Stabilize disease |
| What are your treatment goals in third-line mCRC?^b^  What are your third-line treatment drivers?^b^ | Improve progression-free survival  Limit toxicity  Maintain patient’s autonomy  Maintain performance status  Preserve quality of life  Prolong overall survival  Relieve symptoms  Shrink tumor size  Stabilize disease |
| HER2 = human epidermal growth factor receptor 2; mCRC = metastatic colorectal cancer; MSI = microsatellite instability.  ^a^For each test, the physician had the choice of systematically/in certain patient cases/never or almost never; ^b^For each option, physicians were asked to rate them as first, second, or third choice. | |
